# The genotoxic burden of toxic metals: bridging molecular mechanisms, biomonitoring, and multidisciplinary interventions

**DOI:** 10.3389/ftox.2026.1906308

**Published:** 2026-10-07

**Authors:** Sameera Reem Imthiaz, Suryapratap Ray, Gracy Anu Francis, Rahul Vashishth

**Affiliations:** School of Biosciences and Technology, Vellore Institute of Technology, Vellore, India

**Keywords:** chronic toxicity, environmental contaminants, genotoxicity, heavy metal, toxic elements

## Abstract

We review the genotoxic effects of trace metal exposure and evaluate an efficient treatment strategy. Toxic metal(loid)s cause significant risks to animal health and ecosystems because of their toxicity and bioaccumulation potential. We then examine the mechanisms of genotoxicity, which include direct DNA damage, oxidative stress, epigenetic alterations, and interference with DNA repair mechanisms. This review highlights the health implications of toxic metal exposure: symptoms like increased cancer risks, reproductive and developmental toxicity, and chronic health disorders. Various assessment and biomonitoring techniques for toxic metal genotoxicity, including biomarkers like DNA adducts and chromosomal aberrations, and analytical methods such as the comet assay and PCR-based molecular assays, are discussed. We evaluate treatment strategies, including chelation therapy, antioxidant supplementation, and phytoremediation, emphasizing their efficacy and limitations. The importance of regulatory frameworks, public health interventions, and environmental remediation programs in mitigating toxic metal exposure is also discussed. We identify research gaps in understanding the long-term effects of low-dose chronic exposure and interactions between multiple toxic metals. This comprehensive review contributes to the field by synthesizing current knowledge on toxic metal genotoxicity and treatment strategies. We provide valuable insights for researchers, healthcare professionals, and policymakers toward the development of more effective prevention and intervention strategies. We emphasize challenges and future directions and highlight the importance of continued research in this critical area of public health and environmental science.

## 1 Introduction

The threat of toxic metal accumulation is currently at a peak, as industrial progress accompanies environmental degradation. Toxic metal accumulation is silent yet insidious at a molecular level. “Trace elements” or “toxic metals” refer to metallic elements with relatively high densities, atomic number, and atomic weight. For example, metal(loid)s such as arsenic (As), cadmium (Cd), chromium (Cr), mercury (Hg), lead (Pb), and vanadium (V) fall under this category. These metals are a primary source of concern from an environmental point of view due to their bioaccumulation potential as well as their high toxicity. According to the World Health Organization (WHO), approximately 143,000 deaths annually are attributed to Pb exposure alone (WHO, 2011). Globally, it is estimated that over 1 billion people are exposed to unsafe levels of toxic metals through contaminated water, food, and air (Rees et al., 2020).

Owing to their extensive usage in the industrial, agricultural, technological, and medical sectors, the toxicity spread by these metals has become prevalent due to natural causes as well as human-induced activities such as mining, industrial emissions, and wastewater discharge. Worldwide, more than 20 million people are directly affected by toxic metal contamination from mining. For instance, Cr contamination from tanneries affects water quality in over 30 countries, placing millions at risk. Their continued presence in the environment and infiltration of food chains pose a great risk to human health, ecosystems, and biodiversity, with toxic metal pollution threatening over 100 million people globally—making it one of the world’s worst pollution problems. These elements are also classified as carcinogens by several regulatory organizations.

The toxicity of metals is dependent on the dose and is also influenced by the route of exposure, chemical species, and individual factors such as genetics and nutritional status. When recurring exposure occurs via polluted air, water, and food, toxic metals slowly accumulate in body’s tissues and organs. A study by the Centers for Disease Control and Prevention (CDC) found that in the United States, more than 500,000 children have high Pb levels in their blood (reference value-5 µg/dL) (Wheeler and Brown, 2013). Cellular functions like cell repair, growth, and differentiation are interrupted during these exposures. Reactive oxygen species (ROS) disrupt antioxidant defenses, and several enzymatic functions are also generated. These bind selectively to macromolecules and eventually intensify oxidative stress and damage at the cellular level. The ability to damage genetic material—“genotoxicity” —is the major consequence of chronic toxic metal exposure. In this scenario, these metals will directly interact with the DNA and interfere with its pathways. Trace metals like Cd, As, and Cr are known to cause DNA mutations and induce carcinogenesis. Long-term As exposure is linked to an estimated 140 million people in at least 70 countries whose drinking water contains unsafe As levels (UNICEF & WHO, 2018).

The toxic nature of these trace metals emphasizes the need to develop efficient treatment strategies. Approaches such as chelation therapy and antioxidant supplementation are used, but they also have limitations such as side effects and accessibility challenges. In fields such as gene therapy, phytoremediation, and nanotechnology, more research is needed in order to provide potential treatment solutions. Genotoxicity induced by toxic metals is a growing threat to the environment that must be managed with an integrated approach that combines innovative treatments and effective public health policies.

The present review examines how chronic exposure to trace elements leads to genotoxicity through DNA interaction, ROS generation, and their interference with repair pathways. It examines the epidemiological evidence of global exposure, describes cellular and molecular mechanisms of damage, and critically discusses current management options such as chelation and antioxidant therapy, along with emerging approaches such as phytoremediation, nanotechnology, and gene-based interventions. We argue that toxic-metal-induced genotoxicity is not a biochemical issue but a universal environmental public health problem that requires integrated therapeutic, regulatory, and preventive strategies.

## 2 Mechanisms of genotoxicity induced by toxic metals

### 2.1 Direct DNA damage

Cr and Cd are among the most extensively studied genotoxic metals. They interact with DNA through adduct formation, intra-strand cross-linking, and oxidative base damage (Figure 1). Cd, generally known scientifically as Cd(II), has been observed to easily cross cellular membranes, after which the metal is considered highly toxic. DNA strand breaks and oxidative injuries to DNA bases have been recorded (Jadoon and Malik, 2017), and Cd also causes DNA adduct formation and instigates intra-strand cross-linkages. Zhou et al. (2013) studied Cd-induced DNA damage in human bronchial epithelial cells (16HBE) and demonstrated progressive increases in DNA damage rates from 4.75% in control cells to 49.00% in tumorigenic cells. They revealed that Cd treatment resulted in tail lengths of DNA comets increasing from 12.8 ± 1.76 μm in untransformed cells to 48.5 ± 2.47 μm in tumorigenic cells, with concurrent mutations in DNA repair genes including hMSH2, ERCC1, XRCC1, and hOGG1. ROS are primarily formed in the presence of Cd, which is a significant contributor to oxidative stress in biological systems. Adverse modifications such as single-strand breaks are induced by Cd, while it also increases error-prone trans-lesion synthesis during DNA replication (Jadoon and Malik, 2017). While similar to Cr regarding ROS generation, Cd’s modulation of genomic integrity is slightly distinct. DNA continues to be a main target for these metal-induced processes, which threaten the fidelity of genetic data.

**FIGURE 1:**
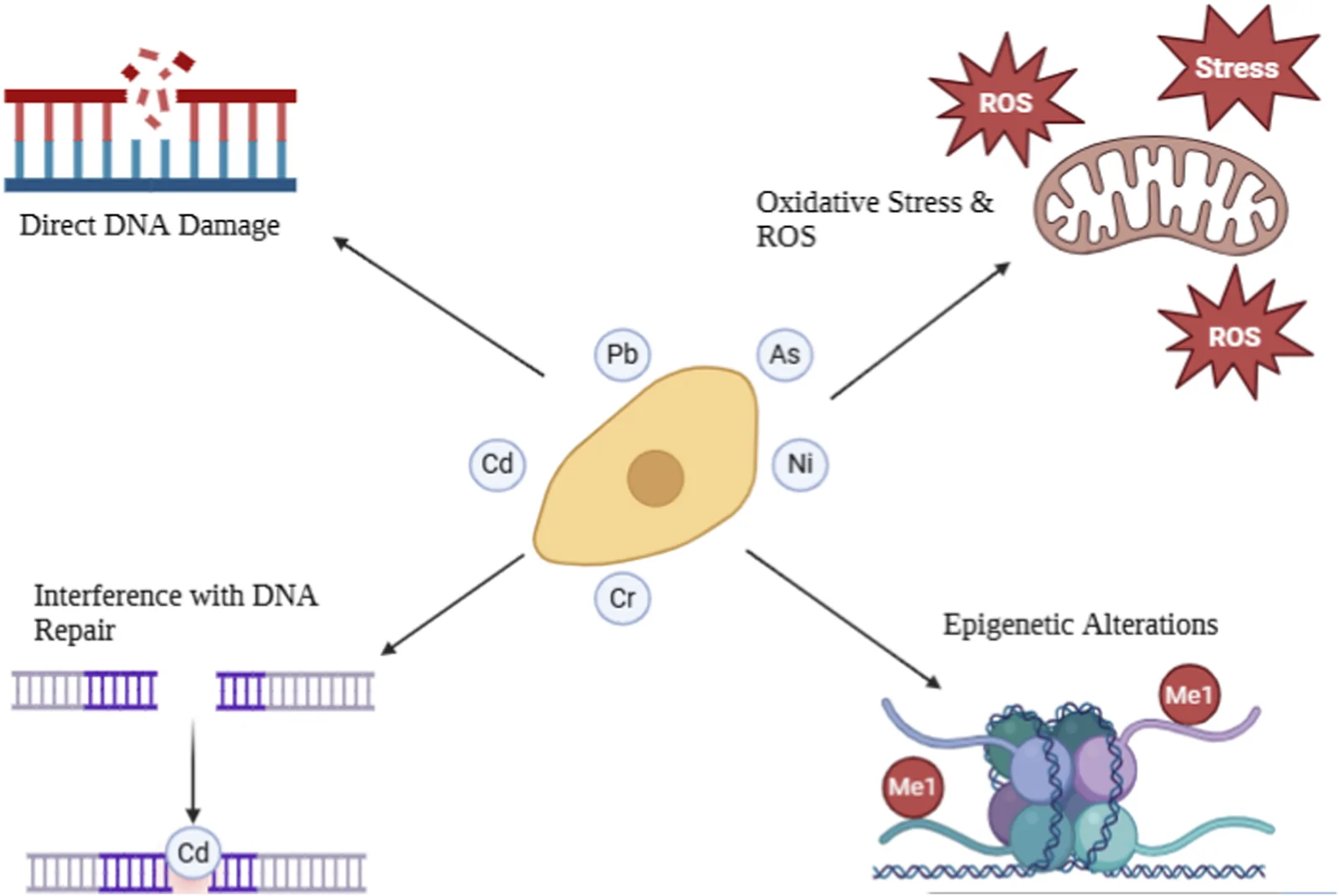
Key routes of metal-induced genotoxicity, direct DNA damage, oxidative stress, interference with DNA repair, and epigenetic alterations.

Toxic metals impede DNA repair mechanisms, making their genotoxic effects even more serious; As, for example, attaches itself to zinc finger motifs, thus blocking DNA repair capacity by lowering it, causing chromosomal aberrations (Kocadal et al., 2020). This reduces genome stability. In addition, changes to the double-strand break (DSB) repair pathways are also produced by these metals. A multi-generational study by Nava-Rivera et al. (2021) demonstrated that chronic As exposure (1 mg As_2_O_3_/L) in rats caused persistent genotoxic damage across four generations (F0–F3). The study revealed that parental As exposure resulted in transgenerational DNA methylation changes and genotoxicity in white blood cells, with reproductive defects persisting even in unexposed offspring generations, thus highlighting the long-term genomic consequences of As-induced DNA damage.

Exposure to Cr and nickel (Ni) has been linked to non-homologous end-joining, known for its error-prone nature, by which mutations are introduced (Morales et al., 2016). The resulting consistent and ongoing genomic changes increase both mutagenicity and carcinogenesis in affected organisms. From a broader perspective, it has been recognized by the research community that such results are influenced by not just dose but also by context, since it changes with factors like the type of cancer present. Such factors are significant. A steady rise in research output has demonstrated the importance of considering toxic metals as part of environmental health challenges. These effects are either amplified or dampened by the circumstances. For example, metallothioneins or antioxidant defense systems may alter the toxic effects of these metals. Possible effects from epigenetic modifications, such as DNA methylation and histone changes, have also been reported by newer research targeting the effects of toxic metals. This adds another dimension to the field of industrial-metal-triggered health. Usage of integrated molecular and epidemiologic approaches is therefore recommended by the prevailing body of research for risk assessment for these environmental contaminants; such approaches are thought to be most useful in light of the wide range of contributing factors.

### 2.2 Oxidative stress and reactive oxygen species (ROS)

ROS cause persistent oxidative stress and DNA damage. This occurs mainly due to the actions of redox-active metals like iron (Fe), copper (Cu), and Cr that participate in Fenton-like reactions, generating molecules such as hydrogen peroxide (H_2_O_2_), hydroxyl radicals (HO·), and superoxide radicals (O_2_^−^), which attack key biomolecules (Ercal et al., 2001; Valko et al., 2005). The response occurs rapidly. Hydroxyl radicals, among the most prominent forms, are capable of inducing DNA changes, cross-links, along with strand breaks Valko et al. (2005), Sinha et al. (2009) conducted a detailed investigation of Cr-induced oxidative stress in *Pistia stratiotes* using multivariate modeling approaches. They exposed plants to hexavalent Cr concentrations (0–160 μM) and demonstrated that malondialdehyde (MDA), superoxide dismutase (SOD), ascorbate peroxidase (APX), and non-protein thiols dominated the oxidative stress response in root tissues, while discriminant analysis identified APX as the key discriminating variable between root and shoot tissue responses. An elevation in hydroxyl radical concentration is usually followed by genomic instability. In this context, antioxidant defenses can be compromised, such as glutathione (GSH), leading to GSH depletion and causing the DNA of cells to be significantly disturbed (Ercal et al., 2001). ROS accumulate faster due to this process. Cells can react differently as key biomolecules are targeted, and this can lead in some cases to loss of functional stability. Such molecular attacks by ROS, driven mostly by generation from toxic metals, often contribute to DNA lesions and genome instability, disrupting normal cellular functions. A similar pattern has been observed in vertebrate models. Velma and Tchounwou (2013) have found that Cr(VI) exposure in goldfish (*Carassius auratus*) induced concentration-dependent oxidative DNA damage and altered antioxidant enzyme activity in the fishes’ liver and kidney tissue. This mirrors the oxidative stress cascade seen in Cr-exposed *P. stratiotes*.

Toxic metals, well-known for their ability to start reactions like the Haber–Weiss reaction, turn superoxide and hydrogen peroxide into hydroxyl radicals (Jadoon and Malik, 2017); such radicals can be hazardous. These metals initiate reactions in which electron-depleting ROS are generated, which damage both nuclear and mitochondrial DNA (Jadoon and Malik, 2017). In addition, toxic metals can produce forms of severe oxidative damage to genetic material. If these oxidative lesions are not repaired, they may lead to mutations such as base modifications and DNA strand breaks, which result in carcinogenesis. The connection between mitochondria and chloroplasts is disrupted by toxic metals, causing excessive ROS production and DNA damage (Sachdev et al., 2021). Impaired growth in plants is closely related to such disruptions.

In addition to previously specified molecular pathways, the degree of oxidative damage caused by toxic metals is influenced by factors such as exposure concentration, time of exposure, and cellular context; observations are important, since variations exist across different biological systems. Certain cell types exhibit higher tolerance. For example, the existence of specialized metallothioneins and phytochelatins in particular plant species allows for the chelation of excessively high levels of organic acid, which may be insufficient under extreme conditions. The persistence of low-level toxic metal exposure, especially in polluted settings, has been indicated as causing DNA damage and eventually increasing the likelihood of malignant change in affected cells. Therefore, when exposure levels are excessive, their impact has been shown to accumulate progressively across time. It was also found that the cellular microenvironment can drastically shift outcomes, and further studies reveal that these complex relationships and the degree of damage induced can depend on multiple variables and parameters. Singh et al. (2022) showed that Cr(VI)-generated ROS via Fenton-like reactions overwhelm antioxidant defenses (catalase, SOD, glutathione peroxidase) during chronic exposure, causing lipid peroxidation and DNA damage.

A growing body of evidence indicates that oxidative stress induced by toxic metals is implicated not only in carcinogenesis but also in a wide spectrum of degenerative diseases, in neurotoxicity, and in disruptions to immune system response, which cannot be underestimated and are supported by increasing experimental data; consequently, neurotoxicity along with immune dysfunction have been linked to the presence of toxic metals in the environment. Different cells and cellular signaling pathways interact in intricate and sometimes unpredictable ways. Genes involved in various inflammatory responses can be upregulated by transcription factors, such as NF-κB and HDAC1. However, ROS in the cellular environment cause random mutations. There are bidirectional effects between them. Toxic metals cause risks such as oxidative and inflammatory responses in biological systems. This is a worst-case scenario that complicates therapeutic approaches, causing major challenges for scientists. Both antioxidant treatment and metal detoxification protocols must be developed within a strategic, multifaceted framework rather than singly.

Studies have shown the application and importance of developing various biomarkers such as 8-oxo-dG concentrations in blood or urine, which can play a role in early monitoring of oxidative DNA damage among individuals at risk for cancer. However, validation remains, even though these methods of detection methods improve early safety measures. These markers enable early detection in communities at risk and those with developmental abnormalities. Trace elements play dual roles as direct stress agents as well as having impacts on antioxidant systems. Understanding these roles is crucial for the improvement of public health policies and drug development, among other things.

### 2.3 Epigenetic alterations

Epigenetic changes due to exposure to trace elements lead to changes such as DNA methylation, histone modification, and miRNA expressions; these shift the usual gene regulation processes and thus cause changes in human pathology (Bitto et al., 2014; Martinez-Zamudio and Ha, 2011). Studies have shown that As exposure induces the hypermethylation of tumor suppressor genes, leading to DNA hypomethylation and thus forming genome instability that has been linked to disruptions in fundamental biological processes. Similarly, alterations caused by Cd and Ni exposure have been found to cause both hypermethylation and the silencing of genes involved in DNA repair and tumor suppression, thus increasing the risk of cancer. Zhang et al. (2024) studied the hepatic cells (THLE-3) that are exposed to toxic metals and found that combined exposures with organic As and CdCl_2_ resulted in the transcriptional repression of H19 lncRNA and the activation of IGF2 mRNA. They demonstrated that significant correlations exist between dysregulated H19/IGF2 expression and hypermethylated CpG sites within the H19 differentially methylated region (DMR), indicating that metal mixtures trigger specific epigenetic aberrations at imprinted gene loci.

Plants exposed to Cd and As also exhibit both the hypo- and hypermethylation of genes connected with metal transport, signaling, and stress responses, making them less capable of detoxifying metal exposure (Chmielowska-Bąk et al., 2023) and thus reducing the plants’ survivability. A comparable dose- and duration-dependent methylation shift has been reported in mammals. Wang et al. (2012) found that low-dose Cd exposure in rats produced widespread hepatic gene hypermethylation alongside reduced apoptosis and increased cell proliferation, suggesting a shared epigenetic mechanism of Cd-induced carcinogenesis across plant and animal systems. The silencing of DNA repair genes was damaging to the organisms’ health. Such findings demonstrate that epigenetic mechanisms play a key role in the biological response to toxic metal contamination and thus must be considered in environmental risk assessments. Notably, parallel transgenerational epigenetic effects have been documented in mammals following chronic As exposure, with altered DNA methylation and genotoxicity persisting across multiple unexposed offspring generations (Nava-Rivera et al., 2021; see Section 2.1). It should be noted that the ability of these metals to induce both global and gene-specific changes in DNA methylation sets the stage for further investigation before being utilized to provide early warning.

Histone mark modification, which affects chromatin structure and function, reacts to environmental exposure to toxic metals, with changes being widely observed in both animals and plants. Activating histone modifications like H3K9AcS10P are increased by As in genes involved in stress response. An increase in gene silencing occurs when repressive histone marks such as H3K9me2P are present, as shown in several experimental models. Indeed, many toxic metals can cause different types of epigenetic change. This is the case with Cu, Cd, and Pb through several mechanisms, such as increased miR-21 expression which contributes to the regulation of protein expression linked to carcinogenesis Bitto et al. (2014), Ryu et al. (2015) also reported similar findings. The disruption of miRNA-mediated pathways involved in metal uptake, chelation, and detoxification has been reported in plants when toxic metals are present, but the details of the regulatory processes are unclear. Recent advances in epigenetics reveal how toxic metal stress imposes long-term changes on plant mitochondria; chronic environmental stress and an increased risk of disease result from such long-lasting molecular changes (Chmielowska-Bąk et al., 2023).

More broadly, the literature shows that the epigenetic landscape is extremely sensitive to environmental toxins which influence gene expression patterns in exposed organisms, so these effects are not just limited to individual lifespans but also influence the stability of specific epigenetic marks which can sometimes be maintained through DNA replication. In the context of environmental pollutants, toxic metals stand out as key modifiers within the epigenome—a factor recorded repeatedly in several recent studies. However, the reversibility of many epigenetic alterations has been described, so that this potential could be realized further as the study proceeds. Epigenetic changes can be transient or reversible.

The relevance of the dose concentration, duration, and the exact developmental stage at which exposure takes place has also been demonstrated, showing that variations in these factors can influence internal epigenetic responses (Bozack et al., 2021; Ray et al., 2014). Exposure during early life compared with exposure during adulthood, more persistent reprogramming of the epigenome, and higher disease susceptibility have been observed, indicating a higher risk profile for younger organisms (Bozack et al., 2021; Kurita et al., 2025; Vaiserman, 2015). These variables must be considered carefully. Risk assessments and regulatory frameworks are required to address these issues, suggesting the need for strict environmental control to be implemented to protect public health and agricultural productivity, though these efforts have not always been maintained and are still under discussion.

Epigenetics increasingly informs crop breeding for metal tolerance, though the functional significance of individual epigenetic marks in plant adaptability remains underexplored. Integrating toxicology, plant biology, and epigenetics will be essential for developing metal-tolerant crop varieties and, more broadly, for translating these mechanistic insights into sustainable agricultural and public health interventions.

### 2.4 Interference with DNA repair mechanisms

Toxic metals interfere with DNA repair mechanisms by inhibiting key repair enzymes. For instance, Cd is known to inhibit multiple DNA repair pathways, such as nucleotide excision repair (NER), non-homologous end-joining (NHEJ), base excision repair (BER), and mismatch repair (MMR), thereby preventing cells from effectively correcting DNA lesions (Morales et al., 2016). Cd and Ni can inhibit the function of the XPA protein, which is important in helping repair bulky DNA adducts and lesions via the NER pathway (Morales et al., 2016). This disruption can impair the ability of cells to remove DNA damage, which can increase the risk of mutations and chromosomal aberrations. Skipper et al. (2016) demonstrated concentration-dependent DNA damage in human liver carcinoma cells (HepG_2_) exposed to CdCl_2_. In their study, comet assay results showed DNA damage increasing from 1.7% in controls to 66.5% at 5 μg/mL Cd exposure, along with increases in comet tail lengths from baseline to 66.5 ± 18.3 μm, providing clear evidence of Cd metal’s ability to over-ride cellular DNA repair mechanisms.

Exposure to As further damages DNA repair mechanisms by inhibiting Poly(ADP-ribose) polymerase-1 (PARP-1), which is a fundamental enzyme and has an important role in the detection and repair of single-strand DNA breaks (Morales et al., 2016). The inhibition of PARP-1 disables the base excision repair pathway, resulting in strand break buildup that leads to double-strand breaks and higher mutagenicity when unrepaired. Failure of these cells to properly repair DNA damage caused by toxic metal interference results in genomic instability. By inhibiting critical DNA repair enzymes and pathways, toxic metals provide conditions for mutations to perpetuate, which leads to carcinogenesis and disease progression.

Broad disruption of DNA repair mechanisms by toxic metals highlights the role of these metals as carcinogens. The persistence of DNA damage is concerning because even minor defects in genome maintenance can increase the mutation load of somatic cells and drive oncogenic transformation over time (Tam et al., 2020). For example, the disruption of the NER pathway not only restricts the removal of adducts induced by exogenous agents but also impairs the repair of endogenous lesions that arise during normal metabolic activity. This suggests that populations exposed to high levels of trace metals such as Cd, Ni, or As are at particular risk, especially if they have genetic susceptibilities in their DNA repair machinery.

Moreover, oxidative stress can be caused by toxic metals in the body, thus further damaging DNA and related repair mechanisms. DNA lesions caused by oxidative stress can be the substrates for the BER pathway, and subsequent BER inhibition by Cd or As increases the accumulation of mutagenic base modifications and abasic sites. This causes double-strand breaks and, when combined with non-homologous end-joining (NHEJ) or homologous recombination, increases the chance of chromosomal rearrangements as well as malignancy. Current research continues to uncover the intricate details of repair inhibition by Pb, Hg, Cd, and other less-studied metals, supporting the consensus that improved monitoring, regulation, and remediation of toxic metal contamination is essential for public health (Hartwig, 2013; Jiang et al., 2019).

Finally, an understanding that DNA repair inhibition underlies much of the toxicity and carcinogenicity of toxic metals has important implications. It suggests the integration of DNA repair capacity biomarkers into risk assessment models and for exploring interventions that might restore or compensate for impaired repair pathways as a preventative or therapeutic strategy against metal-induced diseases.

## 3 Health implications of genotoxicities from toxic metal exposure

### 3.1 Cancer risks

Cr, when present in the hexavalent state Cr(VI), is identified as a cause of various cancers in both the lungs and nasal passages, with significant research indicating that this factor also has bronchial effects (Kocadal et al., 2020; National Cancer Institute NCI, 2024). These processes contribute to genomic instability and tumorigenesis in affected tissues. Lung, prostate, and kidney cancer are all associated with exposure to Cd. Wu et al. (2024) conducted comprehensive genotoxicity studies on Hg compounds using human fetal liver cells (WRL-68). Their findings revealed that HgCl_2_ exposure at 0.5 μM and 5 μM concentrations increased nucleus damage by 60% and 166%, respectively, compared to controls, as measured by comet assay tail-moment quantification. Prolonged 7-day exposure to 0.5 μM resulted in 200% increased nucleus damage, demonstrating the potent carcinogenic potential of Hg through DNA damage mechanisms. The primary mechanism for toxicity has been shown to involve the creation of ROS, suppression of DNA repair enzyme function, and disturbance in calcium metabolism (Kim et al., 2015; Kocadal et al., 2020). Cd mimics elements that can disrupt bacteria signaling, so this disruption can occur without ROS forming. Unchecked cell division may, therefore, result from interference by Cd, although this metal can affect signaling by masquerading as other elements. In particular, the risk of cancer is greatly increased by continued exposure at even low levels, and these metals are subject to wide toxicological research.

Pb exposure has been shown to be related to the occurrence of DNA damage, to the inhibition of certain enzymatic activities, to interference with calcium-dependent cellular processes, and to increasing frequency of chromosomal abnormalities (Kocadal et al., 2020; Nagaraju et al., 2022); more studies continue to clarify all the possible mechanisms involved. Multiple laboratory experiments observed these biochemical alterations. Oxidative stress is induced, DNA repair mechanisms are disrupted, and epigenetic changes are associated with cancers of the skin, breast, and prostate (Ziech et al., 2011). The frequency of cancer in affected populations also plays a significant role in the prevalence of cancer. Toxic metals influence DNA methylation changes, causes genomic instability, and can induce oxidative stress (Khoshakhlagh et al., 2024; Kim et al., 2015). Oxidative damage to DNA becomes more likely in these cases. Current data support the correlation between metal exposure and different cancer types by focusing on molecular disruptions at the cellular level. Hemmaphan and Bordeerat (2022) reviewed extensive evidence of the genotoxic effects of Pb, compiling data from multiple epidemiological studies showing the altered expression of DNA repair genes due to Pb toxicity. The carcinogenic effects of toxic metals are high and are based on both direct and indirect cellular shifts; epigenetic modifications introduced by these metals complicate their harmful effects.

Such effects have been achieved by Cr through the formation of DNA adducts and the induction of oxidative DNA damage, thereby promoting an even more adverse tumorigenic environment. Cd, on the other hand, mimics biologically required ions and has the potential to cause cell growth to become uncontrolled, so that apoptosis can be evaded—all important for the onset of cancer. The interplay between all these systems is very complicated. Implications of such mechanistic diversity make the assessment and management of risks from toxic metal exposure more difficult.

Pb trace elements cause cancer and are studied extensively, since it is associated with the calcium signaling pathway in neuronal tissues. These risks might lead to permanent DNA damage that can cause to genomic instability followed by subsequent genetic diseases. Similarly, metal(loid)s like Ni and As are known to cause epigenetic modifications such as DNA methylation patterns that might help sustain tumor-promoting traits, as well as in future prognosis. These effects cause long-term changes at the molecular level in urban populations and are thus linked strongly to consistent exposure to more than one toxic metal. Exposure to toxic metals influences health and disease prevention strategies, and it must remain a priority in cancer preventive research. On the other hand, occupational as well as environmental exposures must be reduced in all fields. Other measures, such as reducing oxidative stress, repairing DNA, and reversing negative epigenetic modifications, are also important. Such strategies should be studied so that inhibition by toxic metals can be anticipated. The global burden of malignancies related to environmental factors can be lessened by these measures, and cancer rates could decrease.

### 3.2 Reproductive and developmental toxicity

Fertility, prenatal growth, and the health of offspring can be subjected to mechanisms such as oxidative stress, DNA damage, and the disruption of the hormonal system, although the exact biological mechanisms involved are not known. It has been particularly recognized that close connections have been made between Cd exposure and miscarriage and other difficulties during pregnancy (Tchounwou et al., 2012). Reduced birth weight is another prenatal risk. Furthermore, the chances of premature birth are considered higher for the infant, and those risks must be acknowledged in both research and in clinical settings. These associations have been documented in several studies, with Pb also being recognized as posing similar risks of pre-term delivery, although different biological interactions have been proposed at the cellular level. Neurodevelopmental abnormalities have also been found to result from Pb exposure, and such abnormalities have been associated with Pb poisoning (CDC, 2012).

As and antimony (Sb) have been shown to add to reproductive toxicity, and their increased levels in human tissue are linked to recurrent pregnancy loss (RPL) resulting from oxidative DNA damage, although the ability of the cells to neutralize oxidative harm is reduced when these metals are lower than the levels necessary for risk of mutations and anomalies in development. Alrashed et al. (2021) conducted a case–control study that examined DNA damage in females with recurrent pregnancy loss and found significant correlations between toxic-metal-induced oxidative stress and reproductive failure. Their research focused on 50 women with RPL versus control, demonstrating heightened levels of Sb and As in the affected volunteers, with increased levels of oxidative DNA damage markers and low levels of antioxidant-producing capacity.

Hg in its organic form can pass easily through both placental and fetal tissues, causing neurodevelopmental problems and restricting growth (Tchounwou et al., 2012). The accumulation of such metals in fetal tissues has long-term effects on development. From a general scientific perspective, major public health consequences arise from environmental contamination with toxic metals; however, for pregnant women, exposure to such substances is perhaps the most concerning since prolonged exposure—acute as well as chronic—to toxic metals can result in a wide array of reproductive and developmental issues. DNA disruption occurs from oxidative stress and the damage caused by these metals; this disruption is thought by several researchers to serve as the basis of many of the clinical outcomes noted in affected groups. Risk can sometimes extend through time, where these problems are most seen. This broad spectrum of problems shows why attention must be paid to reproductive biology due to possible sample bias, and this may affect the reported associations.

Epidemiological surveys have been widely conducted, and these have continually found a correlation between increased environmental toxic metal exposure and pregnancy complications in both in urban and rural communities. Policy measures should be tightened regarding regulation, and strategies to restrict exposure in the population, particularly among groups such as pregnant women and those intending to conceive, must be implemented firmly. The cumulative effect of exposure is such that, even if absorption is low and occurs on a chronic basis, this exposure has been shown to likely cause negative reproductive and developmental health outcomes with time. It must be stated that the susceptibility of individuals, including their nutrition status, co-exposure to other toxins, and inherited genetic backgrounds, can shift the risk equation and be overlapping. Research suggests that a collective public health stance is to be favored, with the same concerns shared by various groups, and crafting policy approaches to address these concerns is also adaptable in response to regional differences. The adoption of such multipronged approaches should be supported by consensus.

Mechanisms such as oxidative damage, the disturbance of endocrine signaling, and direct toxic damage are the pathways by which toxic metals interfere with fertility and prenatal development; these processes have been the subject of intensive recent research and are of continuing scientific interest. The literature strongly reinforced that addressing exposure to these toxic metals remains important for future generations because significant risk factors are present for populations worldwide. Researchers have recommended ongoing studies and have called for immediate preventive actions to mitigate the impacts of these toxic elements.

### 3.3 Chronic health disorders

Trace elements remain bio-accumulated in the environment and tend to persist indefinitely in nature, causing very serious health impacts, as seen in studies such as Jan et al. (2015) and Tchounwou et al. (2012). Human health is affected by chronic exposure to toxic metals such as As, Cd, Cr, Pb, and Hg, and public health regulators often focus on these. The mechanisms through which toxic metals exert toxic effects are considered multifaceted and very complicated, with oxidative stress, inflammation, mitochondrial malfunction, and disruptions of essential metal ions accepted as mechanisms of damage caused by toxic metals (Jaishankar et al., 2014; Tchounwou et al., 2012). In cardiovascular tissue in particular, the effects of such metals are clear, and many investigations have found that oxidative stress with inflammation contributes to damage of the endothelium, changes to vascular structure, and the progression of both atherosclerosis and hypertension (Jan et al., 2015). Numerous forms of metal toxicity are identified as being generated by toxic metals, and these forms are found within cells and throughout the body (Tchounwou et al., 2012). Blood vessel integrity does not remain intact when these events take place, such as thromboembolic events, greater likelihood of clotting instability, and higher risk of cardiovascular events (Jan et al., 2015). Such factors demonstrate the serious nature of toxic metal exposure.

During certain sensitive stages, such as prenatal development, exposure has been shown to cause more pronounced neurological and immunological effects compared to similar exposures in adults (Jaishankar et al., 2014), and such findings have drawn increased scientific attention. While cases in adults are less severe, developing nervous and immune systems—already multiplying and experiencing elevated metabolic rates—are particularly sensitive (Simon et al., 2015). Vulnerable children are frequently exposed to neurodevelopmental toxicants which may disrupt synaptic transmission, neural differentiation, and cognitive growth, sometimes even at low exposure levels (Jaishankar et al., 2014; Jan et al., 2015). This indicates that effects are still observable at lower levels of toxic metals.

Immune system functions are influenced significantly by chronic exposure to toxic metals, resulting in altered blood cell formation and functions. Not only is there a high incidence of neutropenia and leukopenia, but increased vulnerability to infections; at times, the emergence of autoimmune characteristics may also be observed (Tchounwou et al., 2012). Blood disorders have attracted higher attention. Various studies have reported that the immune system of individuals who are more likely to develop severe allergies is also weakened due to toxic metal exposure (Jaishankar et al., 2014), while some individuals in areas with elevated toxic metal presence are at higher risk due to their continuous environmental contact with these elements. This immune dysregulation elevates cancer risk by impairing the immunosurveillance of malignant cells (Gangemi et al., 2016; Wang et al., 2021) and promoting chronic low-grade inflammation, which favors tumor initiation and progression (Gangemi et al., 2016; Mark, 2025; Shalapour and Karin, 2015). The literature repeatedly calls for early prevention and reduction of exposure to lessen disease prevalence associated with immunosuppression or immune dysregulation (Jan et al., 2015; Tchounwou et al., 2012). Longer exposure time will frequently cause broader systemic effects. These recommendations have been supported by both national and international health authorities.

Generally, toxic metals do not just affect one organ but a wide range of biological systems, and this disruption occurs on a broad physiological scale (Jaishankar et al., 2014; Tchounwou et al., 2012). A number of physiologic processes have been affected by these toxic contaminants, which makes the effects more dangerous and not just limited to the human body. The harms caused by toxic metal exposure can only be reduced through rigorous public health policies, strong regulations, and by research focused on both removal and neutralization methods (Jan et al., 2015). It is believed by authorities that multisectoral pathways should be employed in order to lower exposure and protect human health due to toxic properties of metals such as Pb, As, Cd, and Hg (Jaishankar et al., 2014). The widespread disturbance of physiological functions by toxic metals has been documented across multiple studies, and it has been recognized that human populations should be shielded from the damaging effects of Pb and Cr.

## 4 Assessment and biomonitoring of toxic metal genotoxicity

### 4.1 Biomarkers of genotoxicity

A broader scientific perspective is often needed when studying mechanisms of DNA damage by identifying direct DNA changes such as adducts, oxidative stress measured by 8-OHdG, and chromosome-level injuries like CAs and MN. No single biomarker alone can be considered sufficient for this challenge; scientists generally agree that a broad set of biomarkers, as previously described, can offer a more nuanced and robust picture of cellular response to toxic exposures. The initial molecular contact between toxic agents and DNA is quantified by DNA adducts, while the latter in turn can result from the metabolic activation of DNA. Other characteristics, such as chromosomal aberrations and micronuclei frequency, can be observed to signify either errors in DNA repair or direct chromosomal mis-segregation; these phenomena have been found to precede various types of malignancy. Researchers agree that no individual indicator provides enough information for a detailed assessment. Chromosomal and molecular-level injuries are both important, but a combination of data from multiple biomarkers is necessary for a thorough analysis. If adduct formation occurs early, this may not always be indicated simultaneously. That is why using a biomarker panel is important when studying cellular reactions to toxic substances.

The sensitive and non-invasive nature of these biomarkers has been widely acknowledged, permitting their application not only within occupational settings but extending as well to broader environmental and public health surveillance, thus allowing health professionals and lay people to trust such techniques. The detection of 8-OHdG in urine or blood plasma, which can be performed with minimal procedure, provides a practical and robust method for occupational safety. Additionally, assays focused on chromosomal aberrations and the presence of micronuclei in peripheral blood can support large-scale risk evaluation; valuable data can thus be collected. The sequencing of the genome is important. Future research should prioritize the standardization of these assays and the exploration of their predictive capabilities within risk analyses. The integration of exposure and disease statuses is considered likely to refine risk estimation models. Better protection could then be given to susceptible groups.

### 4.2 Biomonitoring biomarkers

DNA adducts, 8-hydroxy-2′-deoxyguanosine (8-OHdG), and chromosomal aberrations are widely used as biomarkers for genotoxicity, allowing researchers to monitor DNA damage from various toxic metals over different timeframes and in different biological systems. All three biomarkers give reliable clues about the extent of DNA damage and indicate possible cancer risks. Biomarker choice reflects mechanism: 8-OHdG best captures oxidative-stress-driven damage from redox-active metals (Fe, Cu, Cr), while chromosomal aberrations and micronucleus assays better detect clastogenic damage from metals like Cr(VI) and Cd. DNA adducts are produced by metal-bound DNA; this facilitates the identification of carcinogenic events (Kocadal et al., 2020). The main sign of oxidative injury to DNA is 8-OHdG. Exposure of occupational workers to toxic metals has been observed to increase the amount of 8-OHdG, which can act as a biomarker for detecting genotoxic stress (Leelapongwattana and Bordeerat, 2020). DNA adducts, 8-OHdG, and chromosomal aberrations can overlap in their response to genotoxic substances. CAs and micronuclei (MN) generation can be used to detect both numerical and structural damage to chromosomes. Markers like micronuclei identified in peripheral blood lymphocytes can indicate the effects of specific toxicants. Cd, As, and Hg exposures are closely associated with such effects (Kocadal et al., 2020). In individuals who have been exposed to toxic metals, studies have documented reduced control group data. This evidence supports health-risk evaluation (Leelapongwattana and Bordeerat, 2020). The use of these biomarkers for assessing the genotoxic capacity of toxic metals and health consequences over time is largely recognized. Conversely, the relationship among these indicators should be interpreted with attention to confounding variables such as smoking status, occupational co-exposure to other metals, age, dietary antioxidant intake, and genetic polymorphisms in DNA repair genes. These tools have often been applied in occupational health settings, and they play a significant part in tracking the effects of environmental exposure (Figure 2).

**FIGURE 2:**
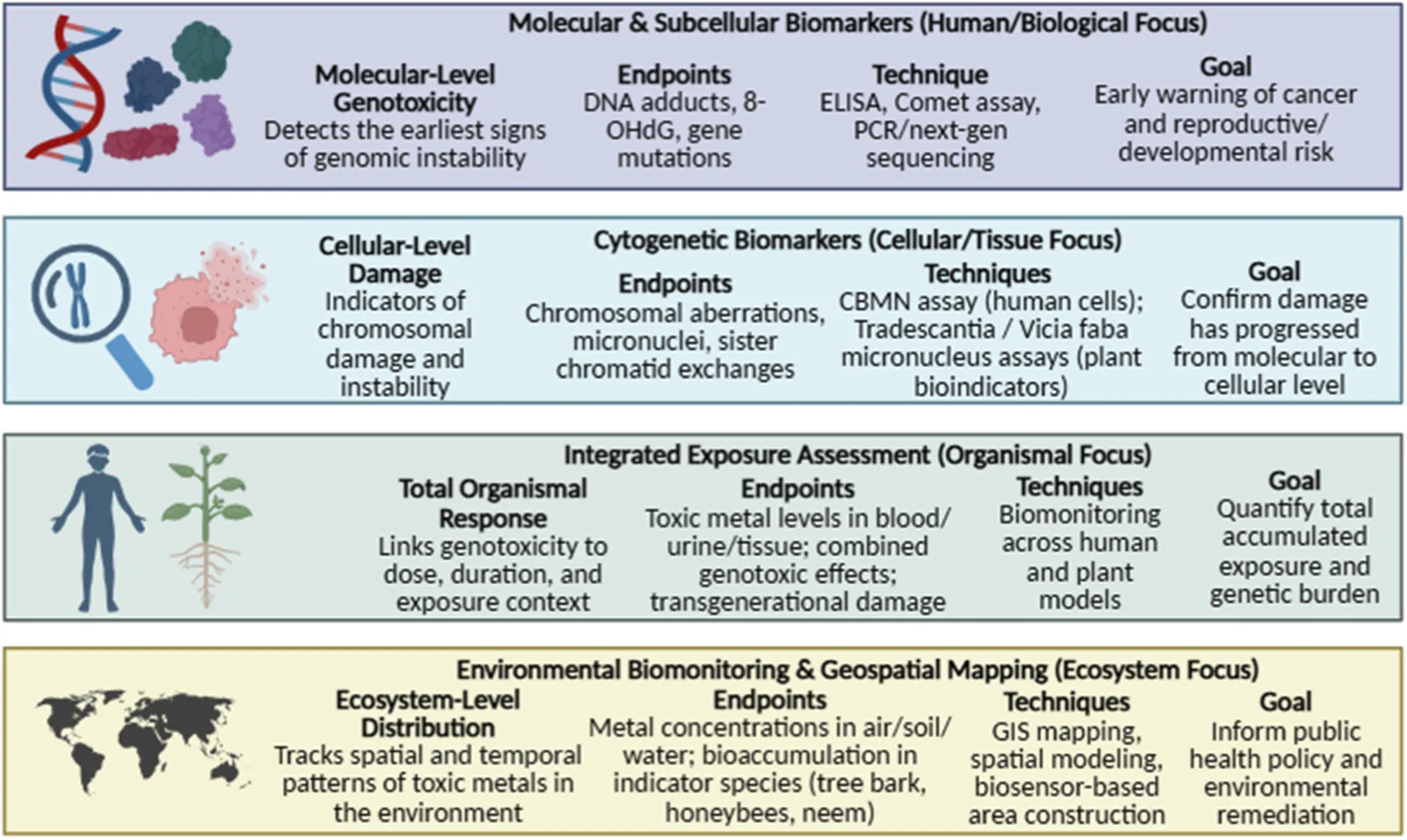
Integrated assessment and biomonitoring framework for toxic metal genotoxicity.

### 4.3 Analytical techniques

Multiple analytical approaches are employed in the measurement of genotoxic effects that target a range of DNA lesions and chromosome modifications, providing information on the presence and nature of cellular damage through different endpoints; the cytokinesis-blocked micronucleus assay represents one of the top protocols in this field for evaluating the frequency of micronuclei within peripheral blood lymphocytes (Leelapongwattana and Bordeerat, 2020) and is widely trusted by researchers. The CBMN assay demonstrates significant worth due to its simple protocol and low cost, but there is also an added benefit: an adequate sample of humans can be obtained efficiently. Its use is widespread across different geographic regions and among diverse groups of people.

Another important tool is the enzyme-linked immunosorbent assay (ELISA) approach, which has been widely adopted for the detection of oxidative damage to DNA (Leelapongwattana and Bordeerat, 2020). This technique provides information relating to environmental exposure; the measurement of 8-OHdG is connected to oxidative stress, which has been increasingly linked to DNA condition. Communities at great risk can benefit markedly from its application, as it accumulates data that show how DNA is altered by oxidation reaction over time and for extended durations. In particular, the method focuses on cumulative changes, making it distinct from point-in-time measurements and allowing researchers to track particular ongoing impacts. It is used to determine the effects of toxic metals.

The comet assay is known as the DNA strand breaks on a molecular scale, and the observations of Morales et al. (2016) confirm that this assay detects significant breaks with exposure to trace elements such as Cd, Ni, and As. Flexibility is a core advantage of this assay and other neutral protocols to target various DNA lesions. Within the environmental genotoxicology, comet assay is very informative as it enables the early detection of DNA damage before visible health effects. However, the sensitivity and variability of the method can lead to inconsistent outcomes. Researchers have been able to adapt the method for different genotoxicants, demonstrating robust utility. The sequence of analysis from ELISA followed by comet assay demonstrates a multifaceted strategy for biomonitoring. Whereas ELISA for 8-OHdG quantifies the aggregate effects on DNA by oxidative stress, the comet assay can provide immediate confirmation.

Methods such as PCR-based molecular assays are commonly used in the therapeutic field because they provide essential information about the mutational effects of toxic metals (Morales et al., 2016). Therefore, when these molecular techniques are combined with next-generation sequencing platforms, the detection of point mutations, insertions, and deletions resulting from faulty repair of metal-triggered DNA lesions is possible across the genome, including low-frequency variants missed by conventional assays. The combination of cytogenetic techniques in this evaluation gives a broad strategy for identifying the intricate mechanisms which may be behind toxic metal genotoxicity. Toxic-metal-induced genotoxicity in plants can be detected by bioassays such as the *Tradescantia* micronucleus (Trad-MN) and *Vicia faba* (Vicia-MN) micronucleus assays. The Trad-MN assay, which uses the DNA of *Tradescantia* plants, measures tissue structure by determining either structural or numerical chromosome changes. The Vicia-MN assay depends on meristematic root samples and indicates exposure through the analysis of micronucleus frequency, which is considered a reliable indicator. Both assays are sensitive and non-destructive, so repeated monitoring is not usually necessary. Trad-MN and Vicia-MN have both demonstrated high sensitivity in the detection of genotoxicity in environments such as metal-contaminated soils and solutions of metal salts, respectively (Knasmuller et al., 1998). Plant-based bioassays, from which many are developed, are specialized since their response time is fast, their application can be scaled, and they detect subtle genetic changes from contaminants. The repeated use of these sensitive species could show cumulative effects, and environments or populations can be monitored efficiently by researchers. Plant-based bioassays hold a significant place, are fast and adaptive, and can detect very small genetic changes.

An integrative and multidisciplinary method that combines cellular, molecular, and biochemical markers will provide the most reliable and precise assessment of the genotoxic risks posed by toxic metals, allowing researchers to capture a broad spectrum of genotoxic effects from each pathway independent of exposure and biological context. The selection of assays must always be guided by the constraints presented by the biological system examined, as well as the nature of the contaminant itself. This review highlights both recent advances and standardized protocols that are critical for assessing and mitigating genotoxic hazards associated with toxic metals as new contaminants and exposure profiles continue to emerge. Specific protocols are being refined as improved analytical techniques become available; protocols must be adjusted so that researchers can stay current.

### 4.4 Environmental and biological monitoring

Biomonitoring programs that detect the levels of toxic metal exposure in populations and across different regions is important. In public health settings and environmental policy decisions, these data are fundamental for developing targeted regulatory and preventative actions. Public health officials and policymakers require the documentation of the systematic measurement of exposure biomarkers. For example, the concentrations of toxic metals that have been used by researchers to identify populations and regions with higher levels of contamination are required for systematic strategy development to minimize this exposure. Longer procedures have occasionally reduced sample throughput, which lowers data resolution and is not ideal in regions affected by pollution. In Nigeria, biomonitoring programs have been merged with geographic information system (GIS) mapping techniques, which have allowed the construction of ecological areas marked by biosensors to be efficiently mapped (Ogli et al., 2023). Similar GIS-based mapping of toxic metal contamination has been applied in Tamil Nadu, India, where spatial interpolation of groundwater sampling data identified contamination hotspots and linked them to elevated health-risk quotients in children, particularly from manganese (Mn) exposure, underscoring the value of GIS in connecting spatial exposure data to biomonitoring-relevant health outcomes (Narayanan et al., 2025). Researchers use these visual representations to identify spatial patterns that typical sampling alone might not catch, and this integration has helped progress effective interventions.

In recent years, combining biomonitoring data with advanced geospatial analysis has increasingly come to attention as a method for improving both the resolution and accuracy of exposure assessments, and this shift has led many scientists to recommend greater integration at the national and international levels. Policymakers have benefited when longitudinal biomonitoring datasets are incorporated by many researchers in order to understand how large and shifting environmental conditions may be affected over time; by building this dynamic temporal element, health advisories and resource allocation are found to adapt more rapidly. This adaptive use is a departure from more static approaches which once predominated. Interestingly, beyond human biomonitoring, environmental biomonitoring has been borne out by plants and tree bark, as well as honeybees, revealing the levels of toxic metal contamination in multiple environmental media, including air, soil, and water (Wacławek et al., 2022; Zavrtnik et al., 2024), as well as within honeybees and their products (honey, pollen, and beeswax) (Zavrtnik et al., 2024). To track toxic metal pollution, tree bark has also been widely adopted as a bioindicator by the research community (Ogli et al., 2023). These methods are regarded as novel monitoring approaches; the use of such bioindicators is highly regarded in the field by many scientists. Notably, bioindicators may reveal contaminants that may be otherwise unavailable if only traditional chemical sampling were used, and this can be particularly important in places where monitoring infrastructure is limited or lacking. Consequently, the practical benefits of utilizing both geospatial techniques and modeling tools become more robust and responsive when these tools are combined. It is clear from several studies that these methodologies allow scientists and policymakers to more precisely address public health problems, regardless of environmental and regulatory conditions.

Recent advances regarding toxic metal exposure can be used with initiatives that integrate environmental and human biomonitoring techniques. These combined methods serve to reinforce the creation of policies and actions that aim to reduce pollution and protect public health effectively. Multidisciplinary collaboration between toxicologists, epidemiologists, ecologists, and data managers in environmental biomonitoring programs has been recommended for meaningful exposure control. Community involvement is seen to be of significant benefit, particularly among residents, which improves data collection, increases public knowledge, and helps build confidence in reported results. Recent efforts have produced technological advances, a collective movement toward integrative strategies, and a marked focus on policy applicability—all vital elements in reducing negative health outcomes from toxic metal exposure. Attention should be paid to ensuring that all regions participate equally, including scientific communities, as they seek to obtain more accurate and scalable data. The active participation of diverse scientific disciplines is still essential, and this cross-field cooperation will probably become more significant if the monitoring of environmental contaminants and their health impacts become more sophisticated, making comparison between data sources more reliable.

## 5 Efficient treatment strategies for genotoxic toxic metal exposure

### 5.1 Chelation therapy

Chelating agents have been widely used to eliminate unwanted substances from the body or in remediation settings, with their uptake by plants during environmental detoxification. The use of these agents in both the medical and environmental fields has seen a history that is shaped by careful considerations of their selectivity and side effects. Risks can arise. CaNa_2_EDTA (calcium disodium ethylenediaminetetraacetic acid) is mainly used as a chelator for Pb poisoning, and it provides significant benefit in situations where acute intervention is necessary. However, in children, decreased reabsorption of trace elements such as zinc (Zn), Fe, and Mn has been associated with repeated high-dose exposure to CaNa_2_EDTA (Zhai et al., 2015). Frequent assessment of kidney function and monitoring trace element levels are thought to minimize the negative effects seen with aggressive chelation, although these protocols are not always strictly adhered to. The issue of metal toxicity (i.e., the pattern of metal ions distributed in biological systems) has encouraged further research into chelating molecules that might display improved selectivity and fewer adverse events. A multidisciplinary management program may include nutrition and supplementation to restore depleted micronutrients, offering a more holistic approach to patient care, and these approaches have become increasingly common. Only with careful application and monitoring can chelation therapy be optimized for each patient. Therapy must be tailored individually.

DMSA (dimercaptosuccinic acid) is a chelation method recommended because it binds Pb and Hg with less harmful effects than EDTA; the preference for DMSA over EDTA in such cases is well supported by recent literature. Gastrointestinal discomforts such as nausea, diarrhea, and food intake reduction were observed by Zhai et al. (2015). Individualized recommendations for patients are particularly significant when a chelation method must be chosen because tolerability can vary. Combination treatments and lower dosing schedules are currently being studied by scientists, who are hopeful that withdrawal symptoms could be reduced. Careful communication and adherence support may decrease treatment drawbacks, as advised by various practitioners. Waidande et al. (2025) evaluated the efficacy of combining chelation therapy with vitamin C supplementation in lead-exposed rats. The combination therapy showed significant reductions in malondialdehyde levels and the restoration of antioxidant enzymes (SOD, catalase) compared with chelation alone. Glutathione levels approaching normal demonstrated the enhanced therapeutic potential of combined approaches. If patients are observed closely, their tolerance to therapy might increase, but sometimes protocols are not completed. Furthermore, it is recognized that the complexity of side effect profiles requires a tailored approach instead of general guidelines. The landmark Trial to Assess Chelation Therapy (TACT) by Lamas et al. (2013) demonstrated the significant clinical benefits of EDTA chelation therapy in 1,708 participants with prior myocardial infarction. This study showed an 18% reduction in combined primary endpoints (HR 0.82, P = 0.035), with 5-year event rates of 32.8% in the chelation group versus 38.5% in placebo; it was found that 18 patients need to be treated to prevent one cardiovascular event.

Present research is focused on safer and more effective ways to reduce harm from metal poisoning, with attempts to reduce toxic effects caused by heavy metals. These methods frequently incorporate antioxidants, vital minerals, and certain plant-derived substances, which have been found to potentially protect body systems from damage and support healing. It has been found that modern molecular engineering makes it possible to develop advanced chelators, including peptide-based structures and nanotechnology-assisted compounds that could provide improved targeting of hazardous metals, while possibly decreasing exposure to them in doses that may be significant. From our perspective, such tactics possess real potential to expand the translation from laboratory research to patient population management, with the realization that understanding the individual genetic makeup and particular biochemical states of persons will allow more precise and safer management strategies to be developed. However, it remains true that scientific translation often encounters obstacles which disrupt progress; hence, careful clinical testing and regulation are required. Reduced off-target effects are especially important for populations with increased susceptibility or those requiring chronic care. Some evidence indicates that therapies should be flexible and specific to support treatment.

The use of synthetic chelators such as EDTA, EGTA (ethylene glycol tetraacetic acid), and DTPA (diethylenetriamine pentaacetic acid) have shown notable results in laboratory and field contexts because they increase the solubility of metals in soil. The use of synthetic chelators causes them to persist in the environment, although this concerns environmentalists due to the possible contamination of ecosystems. The persistence of synthetic chelators in environmental matrices makes it important to balance improved phytoextraction potential against possible widespread chemical contamination. Some compounds with improved characteristics have been proposed as “water chelators” that would break down quickly once metal uptake is enabled, which could mitigate environmental risk. Before the large-scale applications of such agents, environmental scientists stress that detailed risk assessment is necessary. Regulatory and ecological dilemmas also arise in agriculture. This situation requires caution.

Organic chelating agents such as citric, malic, acetic, and oxalic acids are currently being examined as ways to address this problem in environmental cleanup. These acids can remove toxic metal complexes efficiently while also being considered friendly to the environment (Yan et al., 2020); field trials have shown no lasting pollutant residue. Breakdown by micro-organisms can be quickly accomplished as these natural acids are generally produced by either plant roots or naturally occur in field studies with standard protocols, thus causing a notable shift in strategies for sustainable soil cleanup. The intersection of green chemistry and phytoremediation is represented by their use, and a reduction in potential long-term degradation is demonstrated. They are documented to be readily biodegradable by native soil microbes, which contributes further to their environmental profile.

The selection of a chelating agent is affected not only by the metal targeted for removal but also by the detoxification method used for its application, which requires a balance between effectiveness and safety in metal detoxification methods. Future research will involve cross-disciplinary efforts, so input from theoretical chemists should be combined with the expertise of analytical chemists. The latter have contributed many techniques, animal toxicologists and plant biologists offer essential biological data, and bioethicists are vitally important. Increasing the detoxifying potential of chelators while maintaining both human and environmental safety remains a complex aim, but it has always been a core pursuit in the discipline. These factors must be considered fully.

### 5.2 Antioxidant supplementation

Cells can be protected from the damage caused by free radicals by certain molecules known as “antioxidants”, the actions of which are central to counteracting the accumulation of ROS in biological systems. These molecules have been shown to regulate oxidative processes. Vitamin C (ascorbic acid) is one of the exogenous antioxidants that assist in neutralizing the ROS that cause free radical injury (García-Rodríguez et al., 2023) while also helping to prevent lipid peroxidation. The integrity of DNA is at stake without their presence. For instance, free radicals can cause DNA damage from peroxidative effects, whereas vitamin C can scavenge free radicals effectively (Mansoor et al., 2023). Damage would occur without their functions. Glutathione, which is classified as a thiol-based antioxidant, is used for stabilizing ROS states, an action that stops oxidative stress and resulting cellular harm (Valko et al., 2005). Cellular redox balance is then achieved. In addition, these antioxidants help sustain the functionality of enzymatic antioxidants such as lipases. Furthermore, antioxidants are able to detoxify ROS commonly produced by toxic metals (Jadoon and Malik, 2017). Since these antioxidants maintain enzyme activity, the detoxification of free radicals occurs, but if the antioxidant levels fall, oxidative stress can rise sharply. Peguero et al. (2014) analyzed the cardiovascular benefits of chelation therapy enhanced with high-dose oral multivitamins and multiminerals. Their analysis of the TACT trial data revealed that the therapeutic benefit was particularly striking in diabetic patients, with the combination approach showing additive effects beyond standard post-myocardial infarction therapies, thus supporting the synergistic role of antioxidants in toxic metal detoxification.

Within cell physiology, cell fate—whether they thrive or die—is shaped by interactions with antioxidants; the complexity of these interactions continues to be examined in many settings. This interplay is significant. It is widely accepted that although antioxidants made within the body, such as SOD, CAT, and GPx, are principally responsible for cell resistance, they also contribute substantially to systemic capacity to resist environmental pollutants and toxic metals. This function is considered just as important, whereas the ongoing action of ROS, especially when oxidative, persistently influences threaten cellular function. The resilience of cells depends on these details. The reaction between vitamin C and vitamin E, for example, allows vitamin E to regenerate vitamin C in cells. The protective shield surrounding cell membranes is persistently reestablished, contributing, as more researchers point out, to the prevention of chronic disease. The continuous renewal of protection thus takes place. Ensuring antioxidant presence at multiple levels in cells has been challenging. Large fluctuations are seen in measured antioxidant levels, which can obscure interpretations and impact cell survival.

The toxic effects of toxic metals are reduced by antioxidants, which act by blocking their redox cycling and binding to metal ions; in this way, oxidative stress is lessened and cellular damage is limited, while the body ages well as a result. This phenomenon has been demonstrated by vitamin E, a compound that has been found to prevent most oxidative damage mediated by Fe, Cu, and Cd in both *in vitro* experiments and animal models. It is also important in protecting cellular lipids and is frequently referenced in related research for its significant antioxidative capacity. Although some research has proposed that vitamin E is harmful to the cell, it has been indicated that Cu might act as an antioxidant, blocking the peroxidation of lipids without causing increased protein oxidation (Valko et al., 2005). Selenium (Se) and N-acetylcysteine (NAC) have also been tested for their roles as possible treatments for toxic metal toxicity as they release low amounts of metal ions from biological systems (Ercal et al., 2001). Currently, gaps remain regarding the therapeutic potential of those antioxidants, since some mechanisms of action are not yet fully understood. Even though future research with these compounds is promising, it does not universally succeed in all contexts, and many unanswered questions remain about concentration and efficacy.

Recent studies have focused on the role of ROS in cellular responses such as apoptosis, immune responses, and cell proliferation/differentiation which directly depend on oxidative activity.

This must also be considered. Excessive antioxidant supplements might blunt the adaptive responses that are mediated by these ROS-dependent mechanisms and should be provided with caution. A complete nutritional fact sheet as well as the needs of the individual should be formulated in order to take the optimum dose of antioxidants, whether through diet or supplementation. The link between the antioxidant and toxic metal is complex; not only does chelation therapy using agents like NAC or COD require regular monitoring, but it can be disrupted if the therapy itself is not carefully done and monitored. A tailored antioxidant protocol is much needed to balance the therapeutic benefits and risks related to overstimulation. When essential metal levels are not regularly checked, risk can occur. These may affect both the complexity of biochemical interaction and the significance of individual variability, and it is vital that the protocols remain dynamic.

The expanding area of nutrigenomics demonstrates how genetic differences can affect the antioxidant levels which people need and how they will change the activity of antioxidant enzymes, which tend to modify how much the body will be protected inside and from outside exposure. Some of these effects are becoming more widely accepted within the research community. The ongoing interplay between oxidative stress, inflammation, and detoxification processes shows that antioxidants are best recognized as part of a larger regulatory system rather than isolated agents, while future targeting of antioxidant dysfunction involves intervention-target treatment, which is likely to become increasingly important in controlling persistent diseases associated with oxidative and toxic metal pressure. There is much promising research. Some groups recently recommended a special focus on antioxidative pharmacological modulation for therapy. The functions of these extend well beyond the maintenance of redox stability, and practitioners still focus more narrowly on radical scavenging. Antioxidant application in treating toxic metal effects has strong backing in the current literature. Both internal and diet-derived antioxidants make up a fundamental defense mechanism against ROS and various metals, and this is generally an active therapeutic area. New evidence-driven approaches for antioxidant use are thus more necessary than ever. The need for more research remains evident. When therapy was proposed, its necessity arose due to the complexity of oxidative and metal stress links, but the field’s rapid development maintains attention on individualized strategies for each element in modern biomedical research, although many older studies treat each element separately.

### 5.3 Phytoremediation

Phytoremediation techniques can be classified into phytostabilization, phytoextraction, phytovolatalization, and phytofiltration, and each of these methods uses distinct plant-driven processes to manage problematic contaminants. They can be ecofriendly and offer the advantages of reduced cost compared to other remediation methods; they are very effective. Metal-tolerant plants participate in phytostabilization as they are utilized for immobilizing toxic metal contamination into broader components of the ecosystem. It has been shown that hyperaccumulator plant species absorb metals from contaminated soil or water and store these metals in their shoots or leaves so that the biomasses may then be collected and the metal-rich material can be disposed of safely. Toxic metals, including Hg and Se, can be released from vegetation species such as *Typha latifolia* and *Brassica juncea* into the atmosphere where their concentrations might be reduced (Gaur et al., 2021). Khan et al. (2022) conducted a case study on phytoremediation of toxic metals from irrigation water in Hayatabad, Peshawar. They examined two locally available plants, *Arundo donax* and *T. latifolia*, for removing toxic metals from industrial wastewater. This demonstrates the significant uptake and translocation capability of these species and how they could be harvested and transported off-site for beneficial use.

From a broad scientific perspective, the integration of bioremediation is very exciting in the field of environmental biotechnology, and it is often observed that a range of approaches play an important role in not only cleaning polluted sites but also in promoting long-lasting ecosystem management; the mechanical excavation, chemical treatment, or other disruptive processes that are characteristic of conventional remediation usually result in an arguably more wide-ranging approach. Several reports show that phytoremediation not just lowers metal toxicity and bioavailability in the environment but also improves soil structure and fertility, which directly contributes to overall ecological improvement. The success of this method is determined by the plant species and the specific environmental situations.

If a hyperaccumulator is chosen inappropriately for a location with low biomass productivity, the effect of phytoextraction drops notably. Alternatively, when a rapid-growing and high biomass species is grown, the contamination removal rate increases. Phytoremediation is a slow process in plants—this may slow harvesting cycles so that toxic metals are not re-released onto the same site. Countries where finances are limited choose phytoremediation on a large scale because of the cost advantages and significant reduction in environmental pollution. On the other hand, there are disadvantages such as slower remediation rates and long-term planning. The importance of site monitoring also continues throughout the operational lifecycle of the remediation process.

Recent studies in genetic engineering and molecular biology demonstrate the substantial potential of improving the effectiveness of phytoremediation. Researchers are actively modifying the bio-oxidative sequestration and producing plants that can target specific toxic metals with more efficiency. These technologies seem to address a number of limitations and can only be achieved through intensive scientific experiments. Suchkova et al. (2014) performed repeated experiments and reported results from both laboratory and *in situ* phytoremediation experiments at Kharkiv’s wastewater treatment plant sludge fields (126 ha total area). They focused on hyperaccumulator species such as Brassicaceae, Poaceae, and Asteraceae plants, demonstrating the effective removal of trace elements such as Zn, Cu, Ni, Pb, Cr, and Cd from contaminated sewage sludge, with particular success using the *Salix* and *Populus* genera for broad-spectrum pollutant remediation. Phytoremediation, as an interdisciplinary process, integrates plant science, environmental chemistry, and ecological engineering, which is an umbrella term for many related fields. Its practical uses are being broadened by expanding research. Field experiments and continual education are refining strategies and extending the practical use for such phytoremediation techniques for the worldwide reduction of metal contamination. With growing public recognition and rising environmental demands, it can a key element for sustainable contamination management frameworks, and its utility will probably be more recognized in the coming years.

## 6 Preventive strategies and policy recommendations

### 6.1 Regulatory frameworks and exposure limits

The U.S. Environmental Protection Agency (EPA), the International Agency for Research on Cancer (IARC), the Occupational Safety and Health Administration (OSHA), and the CDC, along with the WHO, are regarded as regulatory agencies that play an effective role in handling the health risks connected with toxic metals, and these authorities have established rules that guide both industrial and environmental practice. These regulators are recognized by many national governments, and their standards are adopted. As has been classified by the EPA and IARC as a carcinogen; chronic exposure to it impacts millions of individuals globally, especially in Bangladesh, India, Mexico, and Chile, although contaminated groundwater has primarily driven this public health concern (Tchounwou et al., 2012). It should be recognized that changes in industrial activity and the environment may create new regions at higher risk for new exposures.

Cd has been classified by the IARC as a human carcinogen, and OSHA has established an occupational exposure limit of 5 μg/m^3^ for an 8-h time-weighted average exposure, although studies have argued for lower levels (Arita and Costa, 2009; Lützen et al., 2004; Zhai et al., 2015). This risk still remains. Gupta et al. (2020) evaluated the phyto-genotoxicity of As-contaminated soil from industrial areas, providing critical data for regulatory assessment. They analyzed physico-chemical characteristics and demonstrated significant genotoxic effects in plant bioassays, thus contributing evidence for the need to strengthen current As soil contamination limits in industrial zones. Individual variation, cumulative exposures over time, and the presence of other chemicals in the environment have been identified as factors that can increase health risks. Exposed populations should be protected with a precautionary strategy and ongoing risk assessment. These compounds, due to their significant toxicity, have been identified as human carcinogens by organizations like IARC and the EPA (Tchounwou et al., 2012), raising extra concerns for those living near affected areas; the need for improvements in industrial waste practice and regular environmental monitoring has become more obvious as a result. Professionals have noted that consistent efforts involving industry, public health organizations, and government need to be maintained in order to deal with this continuous hazard. Routine environmental monitoring and proper management are essential in preventing continued Cr(IV) exposure. Mismanagement can result in extensive contamination. Joint collaboration is vital; without it, long-term mitigation cannot be effective.

Pb exposure, still recognized as a serious health concern, especially for children, is a topic addressed by the CDC to reduce risks associated with this metal (Tchounwou et al., 2012). However, the issue persists widely and has led to new epidemiological research which suggests even small Pb concentrations may detrimentally impact health. Intensive prevention strategies, broader screening efforts, and the expansion of campaigns for public education have been advocated by Tchounwou to decrease diseases linked with lead. Hg exposure, according to the WHO, represents a risk for many forms of cancer if not been reduced effectively (Tchounwou et al., 2012), and these hazards can impact many species. The Minamata Convention and related treaties are significant moves toward reducing Hg contamination. Sustained application of these recommendations is required for such interventions to succeed over time, but this has not always been delivered.

Regulatory activities act to set safety criteria (Figure 3), protect public health, and direct treatments for minimize risks. Societies can thus respond to the dangers efficiently, giving special priority to at-risk groups. In light of continuing risk, the need for flexible regulatory systems must be emphasized, and global collaboration and the advancement of interdisciplinary research must be supported, since a static approach would not be effective for adaptable problems. Only when scientific progress is integrated with action-oriented health policies can the complicated obstacles caused by toxic metal toxicity be addressed in a manner that will benefit the whole world. Vulnerable populations will continue to be at risk if action is not taken.

**FIGURE 3:**
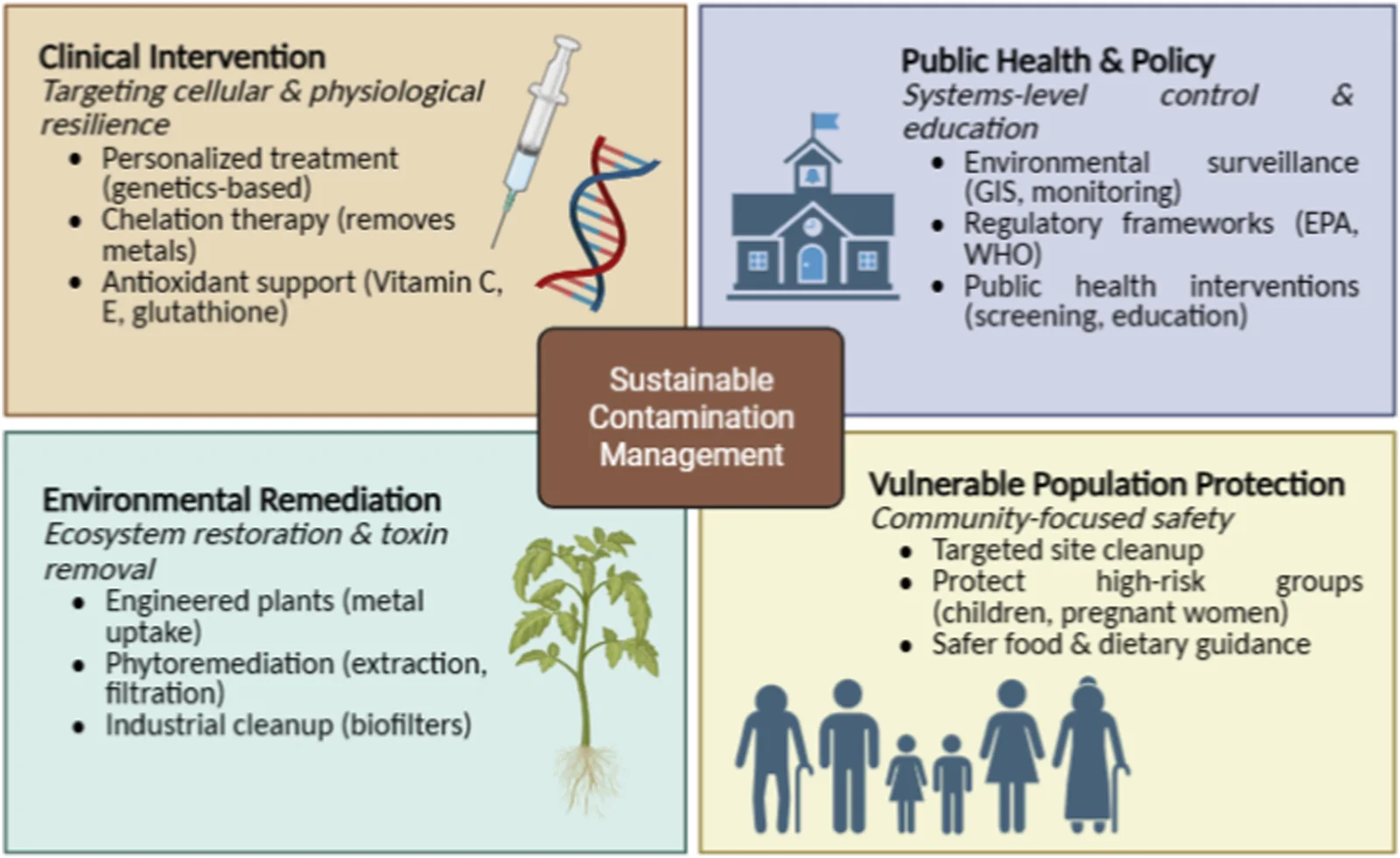
Integrated model for the management and mitigation of toxic metal genotoxicity.

### 6.2 Public health interventions

Efforts to raise awareness about sources of toxic metal contamination, which include contaminated food, ensure that communities may adopt steps that can restrict their exposure to such hazards; participation in educational initiatives regarding safe food choices can lead consumers to avoid items known for high levels of toxic metals, such as Hg in seafood possessing, and dietary recommendations rich in antioxidants, like food containing glutathione, which can improve the overall intake of these nutrients (Bennear et al., 2012). Researchers have recognized that the most important step is to improve health outcomes. Programs in communities that are supported by local health authorities can raise awareness and also promote safer consumption practices. Public health campaigns are intended to spread information about environmental dangers, and their role can be described as educational. Gaps between scientific data and what people do in response to the problem are critical, but everything that happens depends on information. Teaching programs can encourage people to make safer food choices and limit risks associated with dietary exposures, while the dissemination of findings through campaigns increases the acceptance of preventive habits. It should be noted that without active participation from the community, public health efforts could face reduced effectiveness in spreading practices that minimize toxic metal exposure. Each of these strategies, such as education, dietary recommendations, community programs, and health surveillance, demonstrate how a larger response to toxic metals can be expected when they are combined.

Preventive screenings and regulatory measures have been identified as important tools for minimizing health risks, and studies such as the removal of Pb from gasoline and the control of As in drinking water have demonstrated that such approaches can achieve significant reductions in exposure. Early detection and later intervention are supported by routine medical screening for at-risk groups along with the testing of toxic metals in food and drinking water. Dental screening has been used to identify children who are at risk of Pb poisoning, allowing early medical action to be taken (Khoshakhlagh et al., 2024). These will become more apparent as environmental screenings become more common and successful in identifying contamination before it is prevalent. The existing literature supports the perspective that regulatory systems need to keep changing, based on developing findings.

In addition, collaboration between researchers remains pivotal for developing strategies which address both the environmental and human health dimensions of toxic metal toxicity, with researchers frequently advocating for multidisciplinary approaches that bring together toxicology, environmental science, and public health in an integrated manner. Such research requires consistent communication and mutual goal-setting, which is sometimes difficult while research communities are integrating their expertise. Furthermore, the implementation of long-term epidemiological studies and ongoing community health surveillance are often considered necessary to accurately assess the chronic impacts caused by low-level toxic metal exposure, requiring the analysis of affected populations over extended periods. These data have been useful to guide nuanced policy changes and interventions, provided that vulnerable groups can be adequately protected. Lifestyle changes, particularly dietary limitations, can be combined to reduce toxic metal effects, but participation rates in screenings may suffer from a lack of awareness. Choices made at the individual level, strong public health infrastructure, and risk assessments continue to constitute the foundations for successful toxic metal exposure management according to the prevailing scientific literature, as reliance solely on one component is unlikely ensure success. Integrated efforts deliver broader public safeguards.

### 6.3 Environmental remediation programs

The prevention of toxic metals being released into the environment ought to involve improving industrial filtrations and waste control practices, and enacting regulatory action; advanced filtration systems, such as adsorption-based filters and membrane filtration, have been developed where natural, synthetic, and hybrid adsorbents are used, with impressive rates of toxic metal removal (Ayach et al., 2024). These systems, together with precipitation, have also contributed to treating wastewater, although their effectiveness is often restricted by expensive operations, toxic byproduct formation, and their lower performance with some specific toxic metals (Ayach et al., 2024). The installation of biofilters into treatment facilities is recommended by current research because increased toxic metal elimination may be achieved through such systems. Regulatory considerations should not be overlooked. Both regulatory oversight and process integration must be considered carefully, while potential risk from residues or byproducts is an ongoing concern. Regular monitoring is important. The adoption of advanced filtration remains central to modern industrial wastewater management, while the role of monitoring is a dynamic and multifaceted area of ongoing technical progress.

From a broader perspective, it is widely accepted by both researchers and practitioners that a holistic approach spanning multiple sectors and disciplines is needed. Pollution impacts water, soil, and air, so it must be addressed in different ways. Strategies for dealing with such pollution must be recognized by many experts. Conventional treatment methods alone are not enough because the properties of toxic metal pollutants vary greatly with source and environmental conditions. This variety in pollutants makes uniform treatment options less effective. Membrane filtration techniques have been identified as promising, as they can selectively remove contaminants at a molecular level and help prevent secondary pollution—a feature that conventional methods sometimes lack. There have been recent advances in nanotechnology and material sciences, leading to improved membrane filtration capacity and regenerability which are likely to soon transform industrial wastewater treatment. The promise of hybrid adsorbents can be seen in their potential to improve removal efficiency.

Stricter waste management methods have also played an important role in protecting people’s surroundings, and measurable progress has resulted from regulatory steps like banning Pb in gasoline and controlling As levels in drinking water (Khoshakhlagh et al., 2024). While each of these methods is important, combining them with other strategies increases the potential for action. By defining and promoting safe disposal methods in landfill, the leakage of Pb, Cd, and Cr byproducts from industrial and e-waste streams into soil and groundwater can be reduced; biofilters should be used to treat landfill leachate. Consistent monitoring is needed, and source control should always be applied. Monitoring and prevention strategies enable sources of contamination to be tracked and regulated, but sustainable production can significantly reduce the amount of toxic metals released as waste into the environment. Sustainable production also can limit the generation of pollutants. Through the combination of efficient filtration, advanced regulatory application, and improved waste management, it is becoming more feasible to regulate the pollution affecting the environment through these avenues. The environmental benefits of these combined efforts have been observed. Adopting such an approach would help accomplish environmental targets, but periodical adjustments should be considered as policies change and industries adapt.

Many environmental scientists have recommended that community engagement and awareness programs should be considered in addition to traditional practices of pollution management, and these are widely acknowledged. Such programs are intended to raise public awareness of the origins and effects of toxic metal pollution, which may result in industries and consumers adopting more careful practices. This change matters. Real-time monitoring technologies that incorporate IoT and machine learning will also transform pollution management practices, allowing decision-making processes to be based on current data and early detection. Not all companies use these systems now. By adopting this integration, it is possible for industrial operations to always stay within pollution limits, thus reducing damage to ecosystems. The benefits are apparent. A robust approach thus needs advanced monitoring systems, strict waste management practices, and detailed regulatory standards. Recent research has pointed to the need to focus on people and communities in order to maintain safer conditions both for nature and for public health, and this research consistently links effective policy with practical action. Long-term security is achieved by combining these approaches so that populations and the environment can avoid the dangers caused by toxic metals. Sustainable progress has only been made through teamwork at multiple levels. Some strategies still require improvement, but the main steps have been outlined.

## 7 Challenges and future directions

### 7.1 Limitations in current treatment approaches

In the healthcare sector, metal exposure treatments have faced numerous challenges from the side effects of medications. Good results and recovery are not always expected within the population worldwide. Sometimes, minor exposure accumulates over time, causing changes that cannot be reversed, primarily because of existing health issues that might combine with complications from metal exposure. Environmental and health regulations are not always strongly enforced. Health systems in some regions have found it hard to address this rising problem. Recent decades have witnessed a rise in toxic metal contamination, and this has become a significant concern for environmental regulation. When discussing toxicity, it is necessary to consider the ability of toxic metals to stay in the environment and accumulate in living bodies over time, which seriously raises chronic health risks for people who are exposed; this persistence is compounded by resistance to the main medical treatments for removing toxic metals. The main intervention is chelation, but this treatment does come with some risks: CaNa_2_EDTA and DMSA, agents most commonly given for Pb detoxification, may cause renal toxicity and may even lower levels of nutrients, such as Mn; in turn, patients might suffer from nutritional deficiencies (Zhai et al., 2015). Nutritional status might become severe. The risk of depleting micronutrients needed by humans is rarely considered in pretreatment planning, and this neglect can slow a patient’s recovery and possibly compromise long-term health. Clinicians must weigh these tradeoffs carefully when designing therapy protocols.

Appetite loss, nausea, and diarrhea have been reported as side effects of DMSA treatment, which can limit patient compliance and tolerability (Zhai et al., 2015). Such physiological responses are particularly troublesome for those who are already vulnerable, such as elderly individuals and children, due to pre-existing nutrition deficits that may be worsened by such interventions or altered severely by these side effects. It should also be noted that in the current clinical landscape, many regions have poor accessibility and inadequate administration strategies for a considerable number of patients. Monitoring and intravenous infusion are required, and these procedures can be expensive; in some places, access to such monitoring does not exist. Diminished efficacy may result from reduced adherence due to side effects, since patients’ ability to follow prescribed regimens is significantly decreased.

Current disadvantages in treatment for Cd toxicity have highlighted the urgent need for safer and more effective options to address it. No approved safety strategies exist for this agent for broad use. The global population faces significant risk from Cd, largely due to its prevalence in industrial emissions, cigarette smoke, and contaminated food or water sources, but targeted therapies are not currently approved, and clinical needs thus remain unaddressed. Such observations have been made repeatedly by researchers, who found that mobilizing Cd is particularly difficult mechanistically: this metal binds more strongly and thus causes existing therapeutic compounds to be ineffective against its toxicity. Concerns about accessibility and effectiveness are widely considered hindrances to toxic metal detoxification, as effective therapies are not often available in low-income communities.

Chelation protocols require specialist supervision, frequent medical attention, and tend to be expensive, so equity concerns are high. Frequently, those who are most exposed and least economically secure are those who effective options, meaning that the public health risks of exposure remain disproportionately high among the vulnerable. Cardiovascular chelation therapy has demonstrated both the advantages and disadvantages of current approaches. While TACT showed significant benefits with disodium EDTA-based chelation (18% reduction in cardiovascular events), the requirement for 40 supervised infusions over 18 months, costing approximately $5,000 per patient, highlights the accessibility challenges that limit widespread implementation, particularly in low-resource settings. However, managing the toxic metal burden is even more difficult when there are minimum resources to monitor exposures or respond properly to complications during therapy. Thus, medical teams are often subject to injurious exposure. More effective therapies and infrastructure improvements are necessary for groups currently at risk, and failure to address these problems perpetuates health disparities worldwide.

The complete removal of metal deposits cannot be achieved through traditional treatment methods. Moreover, trace metals accumulate slowly in the tissues, causing complications over time. Therefore, predictions based on symptoms cannot be made, and this ongoing tissue accumulation leads to complications not only during the first detoxification stage but also in later stages. However, molecular-level injury can arise from slow responses to intervention; this requires more attention. Antioxidants within dietary supplements are considered a promising potential substitute, since they do not have harmful side effects (Zhai et al., 2015). By neutralizing ROS, which are produced by toxic metals, antioxidants reduce tissue damage and promote internal detoxification processes as described in the literature. While chelation remains the foundational method for treating cases of severe liver disease, supplementation, according to most experts, functions as a practical, accessible and safe supplement to therapy, especially for patients with moderate or mild disease.

### 7.2 Research gaps in genotoxicity mechanisms and treatments

Much research has been performed into toxic metal toxicity, but it is clear that several key domains still demand additional attention. Among these are the prolonged impacts of low-level chronic exposure as well as the interactions involving various exposure mechanisms, which persist across scientific discourse and recommendations (National Research Council, 2000). Most existing studies have been directed at acute toxic impacts, but chronic effects are less clear; while acute toxicity dominates the literature, it has been found that chronic exposure in both groundwater and air may lead to health risks that have not yet been thoroughly described. Considerable evidence links long-term exposure to disorders that affect the cardiovascular, nervous, and immune systems; the mechanisms have not yet been sufficiently clarified. In addition, compounds like Cd, Pb, and As have been categorized as quiet genetic or epigenetic changes triggered by longstanding, minimal exposure, and this continues to provoke scientific debate (Arita and Costa, 2009).

The development of proactive health protocols depends on learning more about transgenerational effects, persistent genetic change, and the formation of illnesses from steady small exposures. Accurately evaluating these extended, low-dose encounters and definite increases is important for scientists. The cumulative nature of these effects can go undetected in the short term, though they may contribute in the future to changing the susceptibility of individuals to the drugs. Research must investigate these subtle but significant changes over longer timeframes to strengthen public health recommendations and regulatory frameworks; effective methodological approaches have not always been utilized. Long-term, low-dose exposure can therefore produce risks that only become visible after many years, with their detection requiring more robust and targeted research strategies. Public health risks come into sharper focus, yet barriers remain in reliably associating low-level, extended metal exposure with specific health problems.

The investigation into the combined effects produced by multiple toxic metals constitutes a significant field that demands careful examination, as most studies have focused only on contaminated cells. Collectively, these exposures might generate synergistic, additive, or antagonistic interactions (Khoshakhlagh et al., 2024); exposure to just one type of metal does not accurately reflect real-life scenarios, so this area requires greater attention. For example, both Cd and Pb have been found to interfere with DNA repair mechanisms, which may result in harmful interactions (Lützen et al., 2004); such interactions have been demonstrated in laboratory settings. The amplification of oxidative stress and epigenetic modifications can also arise through exposure to multiple metals, such as As, Hg, and Cr, the complex effects of which have been detailed by Valko et al. (2005). Increased research efforts must therefore focus on the individual processes as well as their lasting, cumulative health impacts. The study of these effects is endorsed by a growing consensus within the scientific field, since to isolate single-metal toxicity for study is being increasingly recognized as oversimplified. Research in fields related to these exposures will lead to improved risk assessment models, regulations, and more precise treatment protocols. There are many scientists who, through *in vitro* systems, are focused on the effects of combined metal exposures.

The importance of adopting broader research strategies has been noted, with some suggestions that future investigations should integrate environmental, genetic, and epigenetic data for a complete map of human health. In other cases, individual susceptibility factors should be further explored; such factors include the impact of long-term, mixed-metal exposures and could contribute to the development of customized public health strategies. Different laboratories are encouraged to pursue collaborations that will enable the field to address such multi-layered scientific tasks with greater efficiency. Only through collaborations on this scale can the actual social burden be systematically reduced, making outcome measures more reliable.

### 7.3 Technological innovations in treatment and monitoring

Emerging technology has been revolutionizing the early detection and targeted detoxification of toxic metals, as such advances reshape how exposure to these substances occurs in urban environments. This makes monitoring more precise and timely. More precise data have been produced. Biomonitoring, GIS, and bioindicators such as neem bark and honeybees provide real-time information on the scale and extent of metal exposure, proving to be significant in determining contamination levels. However, coverage still needs improvement in certain regions. The slow radial growth and high cation-exchange capacity of neem bark allow it to accumulate metals over long timeframes, making it a reliable long-term indicator, while the wide foraging range of honeybees means that metal residues in honey, pollen, and beeswax reflect contamination across large spatial areas. Researchers have also applied DNA microarray analyses to examine shifts in gene expression from toxic metal exposure, and a molecular level view is underway (Kawata et al., 2007). These techniques allow proactive intervention and targeted remediation efforts by detecting contamination hotspots and exposure risks earlier, which has improved the strategic planning of treatments at varying scales. Research and development continue to advance these technologies, as well as expand public health applications. Targeted remediation has been driven by these innovations, with more effective strategies being developed over time. Detailing such advances, it is clear that a multifaceted approach remains necessary and meets the growing needs of health bodies.

It is often recommend that multidisciplinary methods, which combine environmental science, molecular biology, and data analytics, should be employed for more precise and useful assessments of bioindicator levels at both small and large geographical levels, allowing policy by environmental agencies to be shaped more directly and with greater location specificity. Nanosensors are being developed rapidly, and they have demonstrated great promise for detecting even minute amounts of toxic metals in a wide range of samples such as soil, water, and biological materials. These devices are sometimes portable, allowing health workers and investigators to collect swift and affordable measurements in the field so that data are available at the point of need. Evolving definitions and standards for such technologies become necessary, so the need for rigorous evaluation procedures, such as repeated testing over differing domains, has been discussed widely. Furthermore, rapid detection by nanosensors can provide support for fast decisions but only when reliability has been proven by repeat usage.

In addition to the early identification of cancer risk for most patients, approaches based on DMSA present risks like kidney toxicity and the depletion of necessary metals, issues that have been discussed previously (Zhai et al., 2015). Next-generation chelation therapies aim to address these issues with improved safety and efficacy; increased interest is observed in the development of biocompatible chelators and antioxidant-based approaches, including diets rich in vitamins C and E and probiotics (Ercal et al., 2001; García-Rodríguez et al., 2023). High levels of oxidative damage are important side effects associated with conventional chelators. Environmentally friendly detoxification methods that make use of hyperaccumulator plant species and certain microalgae such as chlorella and spirulina are cited as having promise due to their absorption capacities (Gaur et al., 2021; Yan et al., 2020). Precision biomonitoring systems are now being considered for integration with detoxification technologies, which can increase drug safety by combining chelating enzymes with efficient detoxifying mechanisms to address the problem of toxic metal exposure worldwide. Promising results are being seen in preliminary trials, though longer-term studies are required. Safer practices will be adopted, and improvements in public health will result. Research in detoxification strategies continues to evolve faster than ever.

Wider agreement is developing that future strategies for detoxification should not restrict their scope to only removing toxic metals but must also include the restoration of the physiological balance disturbed by toxicity; this objective makes a broader approach necessary. Endogenous repair systems and the ability of researchers to suffer the negative consequences of these results are now considered. For modern efforts to address contamination, both health improvement and community recovery are now being factored alongside classic removal tactics. Phytoremediation processes by microbial bioremediation help hasten and sustain detoxifying actions *in situ* where these approaches are used. The acceleration of detoxification processes remains a key target; community involvement and government support, together with the scalability of green technology, will probably dictate how successful these approaches are in applications outside laboratory conditions, although more consistent implementation has been called for by practitioners. Environmental sustainability must be combined with human health and technology. The necessity for a multidisciplinary structure, which brings together new technical advances with ecological and health aims, is essential.

## 8 Conclusion

A thorough assessment of chronic exposure to toxic metals and the persistence of these pollutants is lacking in current research; in this context, arsenic, cadmium, chromium, mercury, and lead have been found to possess notable toxicity and potential for accumulation within biological systems, causing DNA damage and interrupting DNA repair processes, all of which contribute to increased cancer susceptibility and problems with reproduction and public health resulting from these persistent pollutants. Health policy must be regularly evaluated, since serious public health impacts are evident. Policy needs have been identified as an area for urgent improvement, and public health strategies including better regulatory approaches and greater biomonitoring are much needed. Science is moving at a rapid pace into the application of chelation therapy and the specific health risks connected to these contaminants, but clear guidance remains lacking. Knowledge gaps about the long-term consequences of exposure to low concentrations and how several metals affect individuals and the community as a whole makes the need for targeted research obvious, even as emerging evidence suggests that increased attention is necessary to develop sound policies based on evidence. Improved science will drive this development, and progress depends on further investigation.

Addressing the complex problems generated by the genotoxic effects of toxic metals requires an approach that integrates multidisciplinary strategies, substantial changes in industrial emissions, and most importantly, proper disposal of waste if meaningful progress is to be made in this field. At the same time, public health education programs must ensure that these measures are applied. The development of more sensitive tools for the early detection of toxic metal exposure should be investigated further, as these tools can detect risks at earlier stages than current biomarkers. Future studies and the development of mitigating strategies must be collaborative. Public health and the reduction of risks linked to continuous toxic metal exposure are best addressed when government stakeholders, researchers, environmental agencies, healthcare workers, and policy regulators work on behalf of each other. By adopting these combined and coordinated approaches, safer living spaces may be attainable, and efforts to improve global health can progress even in the context of widespread contamination.
